# Coffee and tea consumption on the risk of osteoporosis: a meta-analysis

**DOI:** 10.3389/fnut.2025.1559835

**Published:** 2025-03-04

**Authors:** Wopei Li, Yujiao Xie, Lei Jiang

**Affiliations:** ^1^College of Rehabilitation Medicine, Shandong University of Traditional Chinese Medicine, Jinan, China; ^2^Department of Rehabilitation, Affiliated Hospital of Shandong University of Traditional Chinese Medicine, Jinan, China

**Keywords:** coffee consumption, tea consumption, osteoporosis, meta-analysis, risk

## Abstract

**Objectives:**

This meta-analysis aims to quantify the relationship between coffee and tea consumption and the risk of osteoporosis and explore whether such consumption positively or negatively impacts this risk, thereby providing a scientific basis for understanding the effects of coffee and tea on bone health.

**Methods:**

We systematically searched PubMed, the Cochrane Library, and Embase for observational studies published up to November 5, 2024, using medical subject headings (MeSH) and keywords related to “osteoporosis, tea, and coffee.” Statistical analyses were conducted using Stata software version 14.0. A fixed-effects model was used when heterogeneity was low (*I*^2^ ≤ 50% and *p* > 0.1). A random-effects model was used for greater heterogeneity (*I*^2^ > 50%). Publication bias was assessed using funnel plots and Egger’s regression tests.

**Results:**

This meta-analysis included 14 observational studies comprising 562,838 participants published between 2008 and 2024. The pooled analysis showed that coffee consumption is significantly associated with a reduced risk of osteoporosis (odds ratio [OR] = 0.79, 95% confidence interval [CI]: 0.73–0.84, *I*^2^ = 28.9%, *p* < 0.05). Tea consumption also demonstrated a protective effect, with a lower risk of osteoporosis (OR = 0.75, 95% CI: 0.62–0.91, *I*^2^ = 80.4%, *p* < 0.05). Subgroup analysis revealed that high-frequency coffee consumption (more than one cup per day) was associated with a greater reduction in osteoporosis risk (OR = 0.83, 95% CI: 0.74–0.93, *p* = 0.001) compared to low-frequency consumption (less than one cup per day), which showed no statistically significant reduction (OR = 0.86, 95% CI: 0.68–1.07, *p* = 0.171). Similarly, high-frequency tea consumption (more than four times per week) exhibited a slightly stronger protective effect against osteoporosis compared to low-frequency consumption (OR = 0.82, 95% CI: 0.70–0.97, *p* = 0.02).

**Conclusion:**

This meta-analysis suggests that long-term coffee and tea consumption is associated with a reduced risk of osteoporosis. Moreover, a higher frequency of consumption within a moderate range appeared to enhance the protective effect against osteoporosis.

**Systematic review registration:**

https://www.crd.york.ac.uk/prospero/display_record.php?ID=CRD42024612101, PROSPERO CRD42024612101.

## Introduction

Osteoporosis is a metabolic bone disorder characterized by reduced bone density and compromised bone structure, resulting in increased fragility and fracture risk (1). Osteoporosis affects approximately 18.3% of the global population and is a major health concern (2). Advances in molecular neurology, clinical pathology, and diagnostic markers have enhanced our understanding of osteoporosis (3). Environmental and lifestyle factors, including vitamin D deficiency, smoking, alcohol consumption, inadequate calcium intake, high-protein diets, and physical inactivity, contribute to its development (4). However, the role of coffee and tea in osteoporosis remains controversial.

Coffee, a major global commodity with an annual production exceeding 9.2 million tons (5), is a significant source of antioxidants and offers various health benefits such as anti-inflammatory and anti-cancer effects (6). However, excessive consumption has been linked to adverse health outcomes including high blood pressure, seizures, sleep disturbances, and potentially osteoporosis (7). Tea, the second most widely consumed beverage globally (8), also has antioxidant, anti-cancer, and anti-obesity benefits (9). However, its effect on osteoporosis risk remains unclear.

Bone mineral density (BMD) is a key diagnostic criterion for osteoporosis, defined by the World Health Organization as a BMD T-score ≤ −2.5 at the hip or lumbar spine (10). Although some studies have suggested no significant link between coffee or tea consumption and BMD (11), the relationship between these beverages and osteoporotic fractures, which are key outcomes of osteoporosis, remains inconclusive. Research has shown no clear association between tea consumption and fracture risk (12), and evidence of a causal role of tea in osteoporosis is lacking (13). Caffeine in coffee may exacerbate osteoporosis, warranting further investigation into its impact (14). Conversely, some studies have suggested potential benefits of coffee and tea. A 2017 meta-analysis found that tea may reduce the risk of osteoporosis (15), whereas a 2022 study indicated that higher coffee intake may lower the risk of hip fractures in individuals with osteoporosis (16). Most previous meta-analyses have focused on low BMD or post-fracture outcomes rather than pure osteoporosis (T-score < −2.5). Therefore, we conducted an updated systematic review and meta-analysis to further clarify the association between coffee and tea intake and the risk of osteoporosis.

## Methods

This meta-analysis was conducted in accordance with the Preferred Reporting Items for Systematic Reviews and Meta-Analyses (PRISMA) guidelines (Supplementary file S1) (17). The study protocol was registered in the International Prospective Register of Systematic Reviews (PROSPERO) under registration number CRD42024612101.

### Data sources and searches

We searched PubMed, the Cochrane Library, and Embase from their inception to November 5, 2024, without language restrictions. Our search terms included Medical Subject Headings (MeSH) terms and free words such as tea, coffee, and osteoporosis. Detailed search strategies for each database are provided in Supplementary file S2. Additionally, reference lists of the included studies and relevant meta-analyses were reviewed for additional studies.

### Eligibility criteria

Studies were considered eligible for inclusion if they (a) included individuals diagnosed with osteoporosis defined according to the World Health Organization (WHO) criteria, with a BMD T-score ≤ −2.5, (b) assessed long-term consumption of coffee or tea, (c) included individuals who had never consumed coffee or tea for comparison, (d) analyzed the risk of osteoporosis as the primary outcome with effect estimates such as odds ratios (ORs), relative risks (RRs), or hazard ratios (HRs), and (e) were observational studies. Studies were excluded if osteoporosis was not the primary outcome (e.g., those combining low bone density or osteoporotic fractures) or specific effect sizes were not reported. Reviews, conference abstracts, duplicate publications, and studies that lacked relevant results were also excluded.

### Study selection

Two reviewers (WPL and YJX) independently screened the studies for eligibility, first by reviewing titles and abstracts to exclude duplicates and irrelevant studies. Full-text articles were then assessed to confirm eligibility. Disagreements were resolved through consultation with a third reviewer (LJ) to ensure impartiality.

### Data extraction

Data were independently extracted by two reviewers (WPL and YJX) using predesigned forms aligned with the systematic review guidelines (18). The extracted data included the first author, publication year, study design, sample size, follow-up duration, participant age, osteoporosis diagnosis, tea or coffee consumption frequency, and adjusted confounders. Disagreements were resolved by discussion and consensus.

### Risk of bias assessment

Quality assessment for cohort and case–control studies was conducted using the Newcastle-Ottawa Scale (NOS) (19), a tool designed to measure the risk of bias in nonrandomized studies. It assigns a score ranging from 0 to 9 based on aspects such as participant selection, exposure measurement, outcome assessment, and adequacy of follow-up, with scores of 0–3, 4–6, and 7–9 indicating low, moderate, and high quality, respectively. For cross-sectional studies, we applied the AHRQ (20) quality assessment criteria consisting of 11 items, where each item is scored as “1” if the answer is “yes” and “0” if the answer is “unclear” or “no.” The total score was categorized as low (0–3 points), moderate (4–7 points), or high (8–11 points) quality (21).

### Statistical analysis

Data analysis was performed using Stata 14.0. Adjusted ORs (and their 95% CIs) were extracted to evaluate the association between tea and coffee consumption and osteoporosis risk. Heterogeneity was assessed using the χ^2^ test and I^2^ statistic. A fixed-effects model was applied for *I*^2^ ≤ 50% and *p* > 0.1, indicating low heterogeneity. A random-effects model was used for *I*^2^ > 50%, indicating substantial heterogeneity. Sensitivity analyses were performed to assess the robustness of the results. Publication bias was evaluated using funnel plots and Egger’s regression tests. Subgroup analyses, categorized by different study designs and varying levels of consumption, were conducted to further interpret and validate the conclusions.

## Results

### Literature search

**Figure 1:**
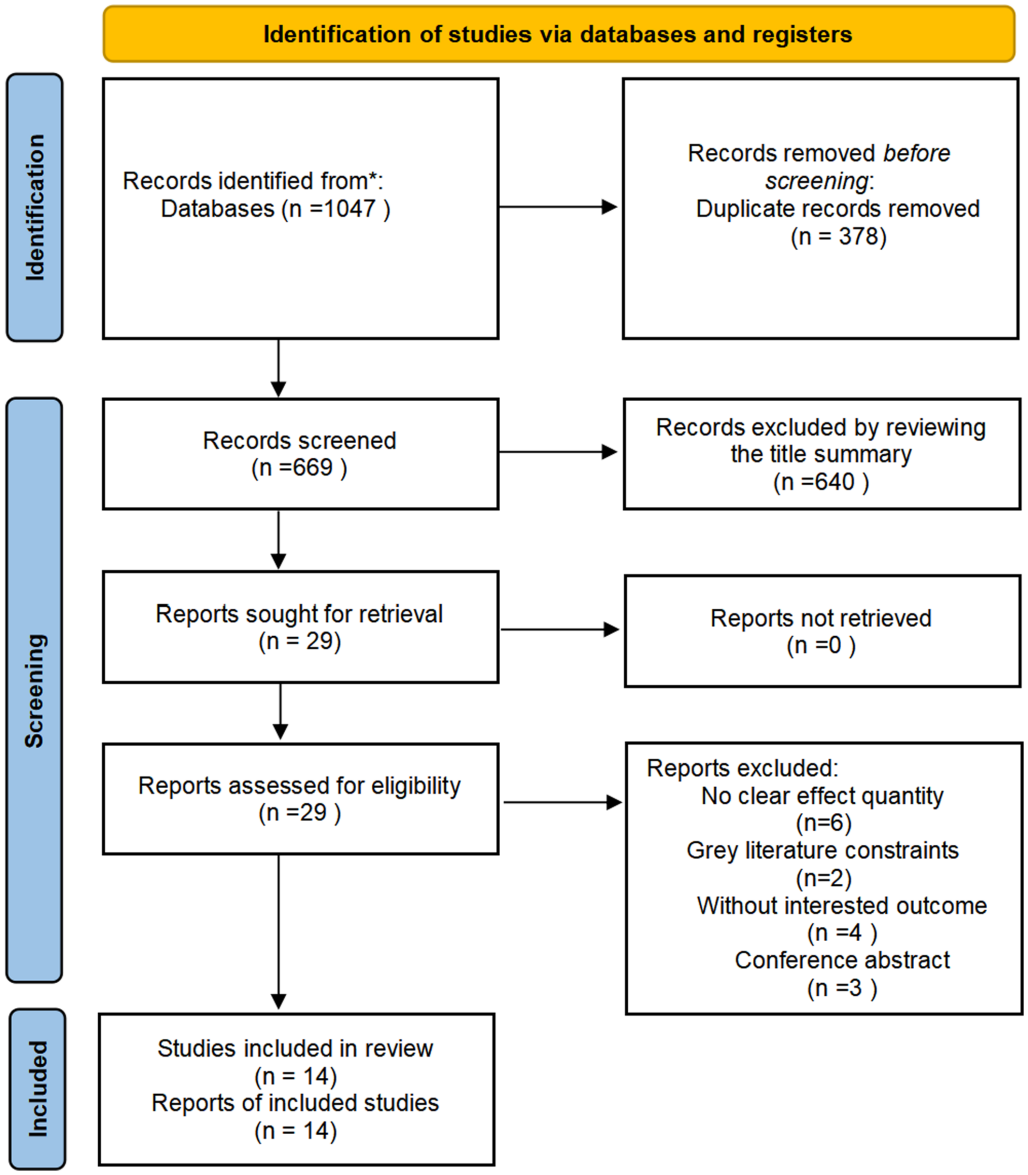
Study screening process.

Literature screening was performed according to the PRISMA guidelines (17). A multi-database search identified 1,047 records (396 from PubMed, 607 from Embase, and 44 from the Cochrane Library). After removing 378 duplicates, the two reviewers screened the remaining 669 records using Zotero. Based on the predefined criteria, 29 records were selected for in-depth evaluation. Nine studies were excluded: six for unclear effect sizes, two for inaccessible full texts, four for lacking relevant outcomes, and three because they were conference abstracts. Ultimately, 14 studies (22–35) met all the criteria to be included in the meta-analysis (five examined the impact of both tea and coffee consumption on osteoporosis incidence, three assessed tea consumption alone, and six focused on coffee consumption). The selection process is illustrated in Figure 1.

### Study characteristics

The included observational studies consisted of 14 studies, encompassing 562,838 participants aged 40 years or older. All studies used clear diagnostic criteria for osteoporosis. Adjusted estimates were available for almost all the studies, although the specific confounders varied. The key characteristics of this study are listed in Table 1.

**Table 1:** Basic characteristics of the included studies.

Author	Year	Country	Study type	Sample size	Follow-up year study period, y	Age (years)	Diagnosis of osteoporosis	Confounders adjusted	NOS/AHRQ scores
Zhang et al.	2024	China	Cohort study	Total: 487,594 OP: 15,211	12.8 averages	56.9	ICD-10	Gender, age, race, BMI, IMD, smoking, alcohol consumption, fruit and vegetable intake, vitamin intake, mineral intake, and physical activity	7
Yin et al.	2024	China	Cross-sectional study	Total: 2,175 OP: 998	2021(7)-2022(12)	OP: 68.73 ± 6.34 NO OP: 72.47 ± 6.89	T-scores ≤ −2.5	NE	8
Xie et al.	2024	China	Cross-sectional study	Total: 2,941 OP: 871	2015(6)-2021(8)	62.48 ± 7.94	T-scores ≤ −2.5	Vegetable oil, animal oil, and cereal consumption	6
Huang et al.	2022	China	Cohort study	Total: 42,742 OP: 3,278	8.5 averages	No tea consumption: 57.8 ± 8.1 Low tea consumption: 54.1 ± 7.0 High tea consumption: 54.6 ± 7.2	T-scores ≤ −2.5	Sex, age, body-mass index, physical activity, smoking status, drinking status, education, diabetes, hypertension, hyperlipidemia, coronary heart disease, stroke, and cancer	8
C.-L. Wu	2022	China	Cross-sectional study	Total: 6,676 OP: 515	2016–2019	No OP: 53.068 ± 0.130 OP: 61.324 ± 0.361	T-scores ≤ −2.5	Tea consumption and vegetarian diet	7
Hao Chai	2021	China	Case–control study	Total: 2,039 OP: 678	2007(1)-2019(10)	62.56 ± 6.67	T-scores ≤ −2.5	NE	6
Li et al.	2021	China	Cross-sectional study	Total: 947	2013(10)-2019(10)	NE	T-scores ≤ −2.5	NE	7
Sunmin Park	2020	Korea	Cohort study	Total: 8,845 OP: 1,136	2001–2002	No OP: 51.3 ± 8.7 OP: 58.5 ± 7.5	T-scores ≤ −2.5	Area of residence, sex, age, BMI, smoking, coffee and alcohol intake, physical activity, and intake of energy, fat, protein, carbohydrates, and calcium	6
Wang et al.	2019	China	Cross-sectional study	Total: 695 OP: 298	2012–2014	67	T-scores ≤ −2.5	NE	7
Yu et al.	2016	China	Cross-sectional study	Total: 992 OP: 90	2011–2014	64.85 ± 9.41	T-scores ≤ −2.5	Age, smoking, alcohol intake, exercise, and medical history	8
Eunjoo Choi	2016	Korea	Cross-sectional study	Total: 4,066	2008–2011	62.6	T-scores ≤ −2.5	Behavioral, diet, factors, age, BMI, monthly, household, Income, education level, hormonal factors	10
Yang	2015	China	Cross-sectional study	Total: 1,817 OP: 524	2011–2014	62.51 ± 9.02	T-scores ≤ −2.5	Age, smoking, alcohol intake, education, exercise, medical and therapy history	6
Abdellah El Maghraoui	2010	Moroccan	Cross-sectional study	Total: 592 OP: 52	NE	49.1 ± 17.2	T-scores ≤ −2.5	NE	7
Afsaneh Keramat	2008	Iran	Case–control study	Total: 717 OP: 381	2002–2005	No OP: 58.2 ± 7.1 OP: 55.7 ± 6.0	T-scores ≤ −2.5	Age, height and weight	6

### Quality assessment

Based on the NOS and AHRQ scoring systems, the average quality score of the included studies was 7.07, with all studies exceeding the threshold of 7, indicating high methodological quality (Table 1).

### Coffee consumption and risk of osteoporosis

**Figure 2:**
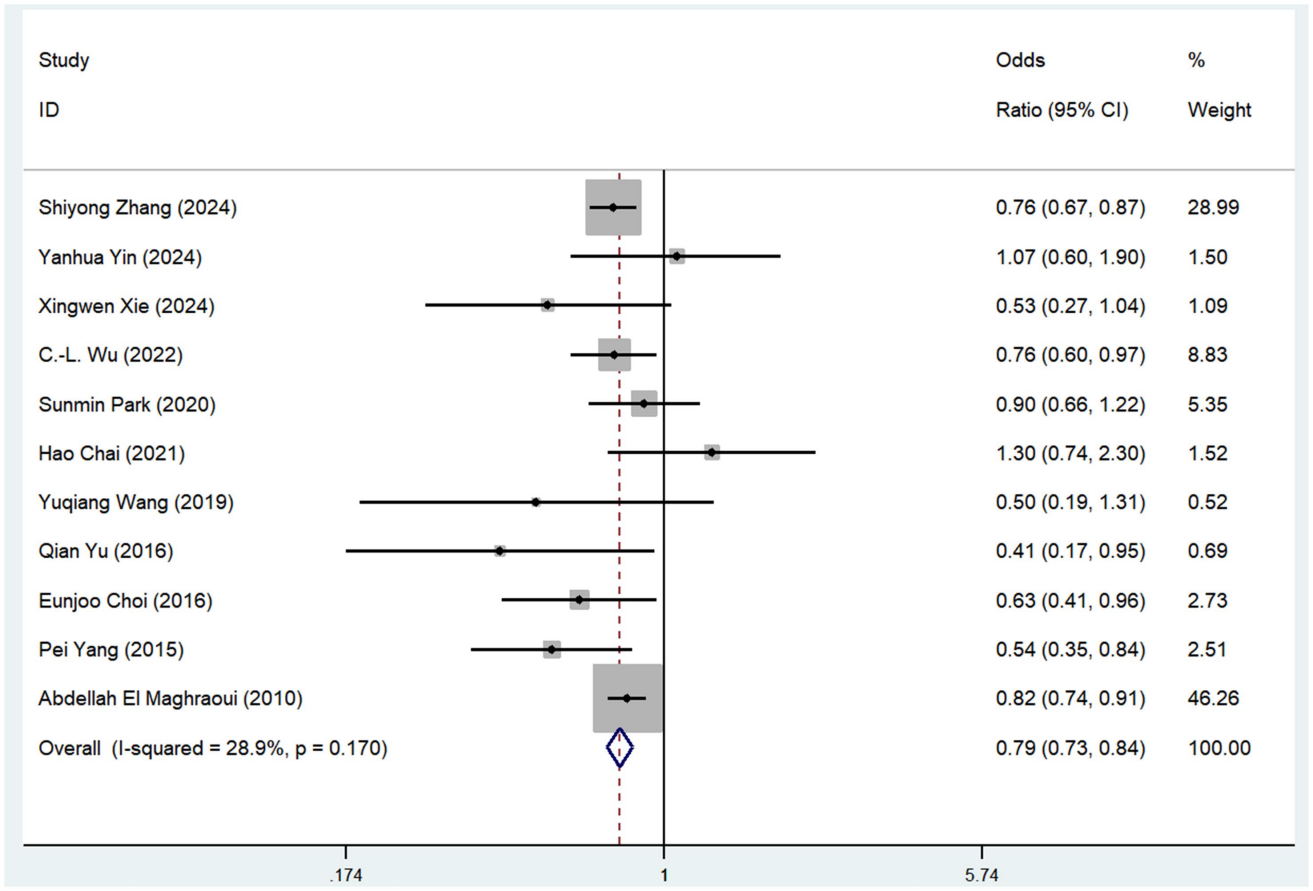
Meta-analysis of the risk of osteoporosis associated with coffee consumption.

Eleven observational studies investigated the relationship between coffee consumption and risk of osteoporosis. The pooled analysis revealed that coffee consumption was associated with a reduced risk of osteoporosis (OR = 0.79, 95% CI: 0.73–0.84, *I*^2^ = 28.9%, *p* < 0.05; Figure 2). Sensitivity analysis revealed that no single study significantly altered the magnitude of the pooled effect, confirming the robustness of the results (Supplementary file S3).

### Tea consumption and risk of osteoporosis

**Figure 3:**
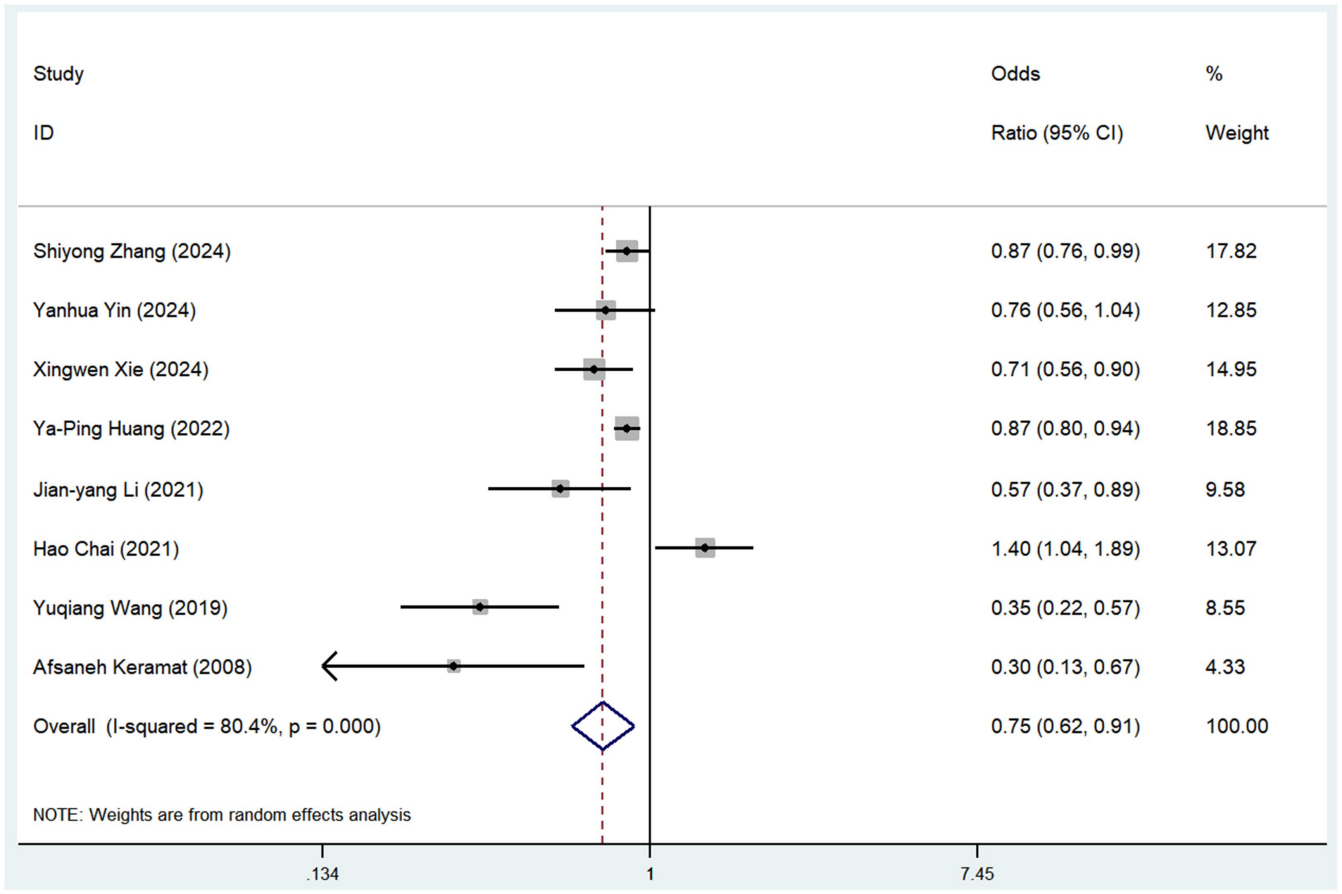
Meta-analysis of the risk of osteoporosis associated with tea consumption.

Eight studies examined the association between tea consumption and risk of osteoporosis (Figure 3). The pooled analysis showed that tea consumption was linked to a reduced risk of osteoporosis (OR = 0.75, 95% CI: 0.62–0.91, *I*^2^ = 80.4%, *p* < 0.05; Figure 3). The sensitivity analysis indicated that no individual study reversed the direction of the effect, supporting the robustness of these findings (Supplementary file S4).

### Subgroup analysis

**Figure 4:**
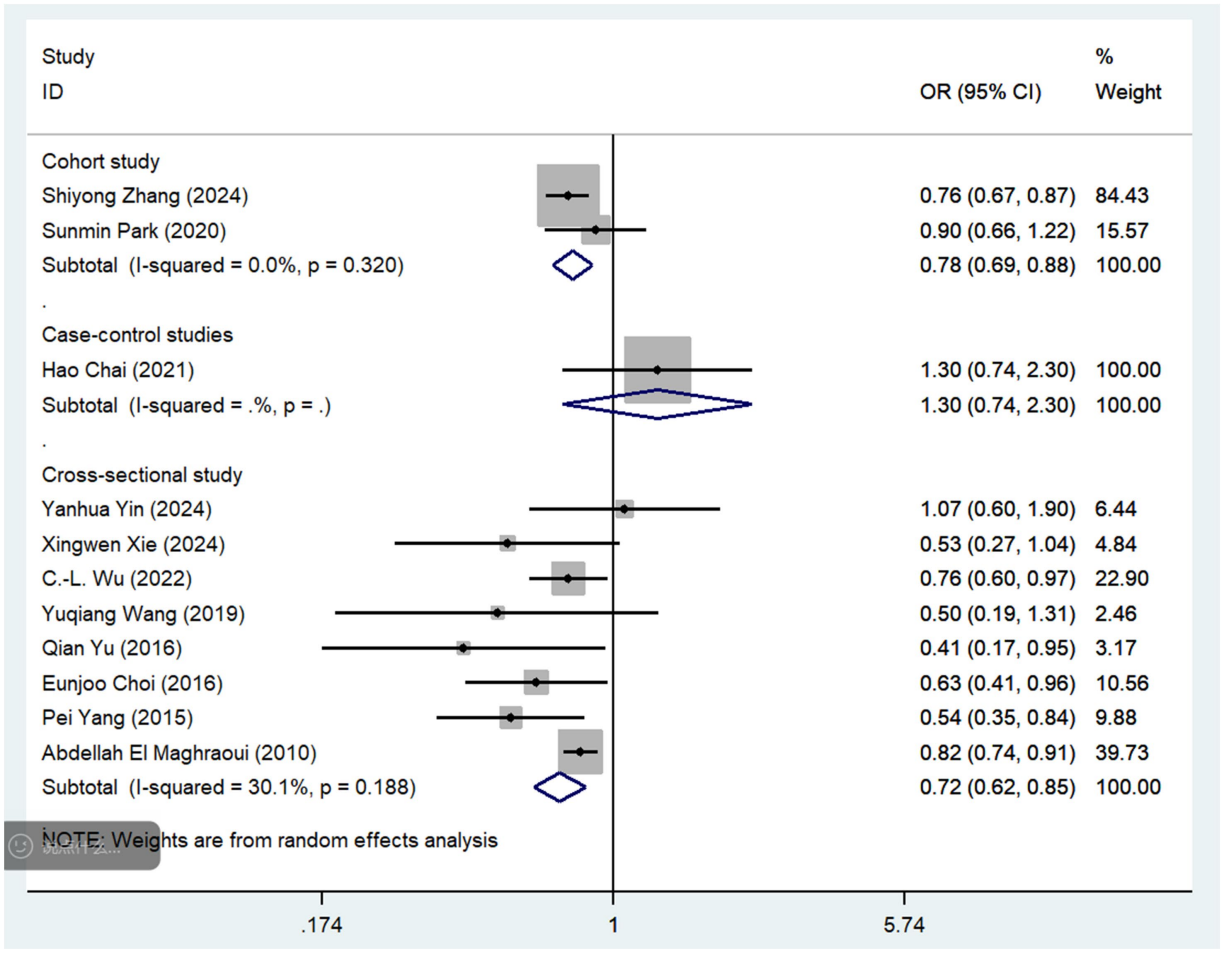
Subgroup analysis of different study types regarding coffee consumption.

**Figure 5:**
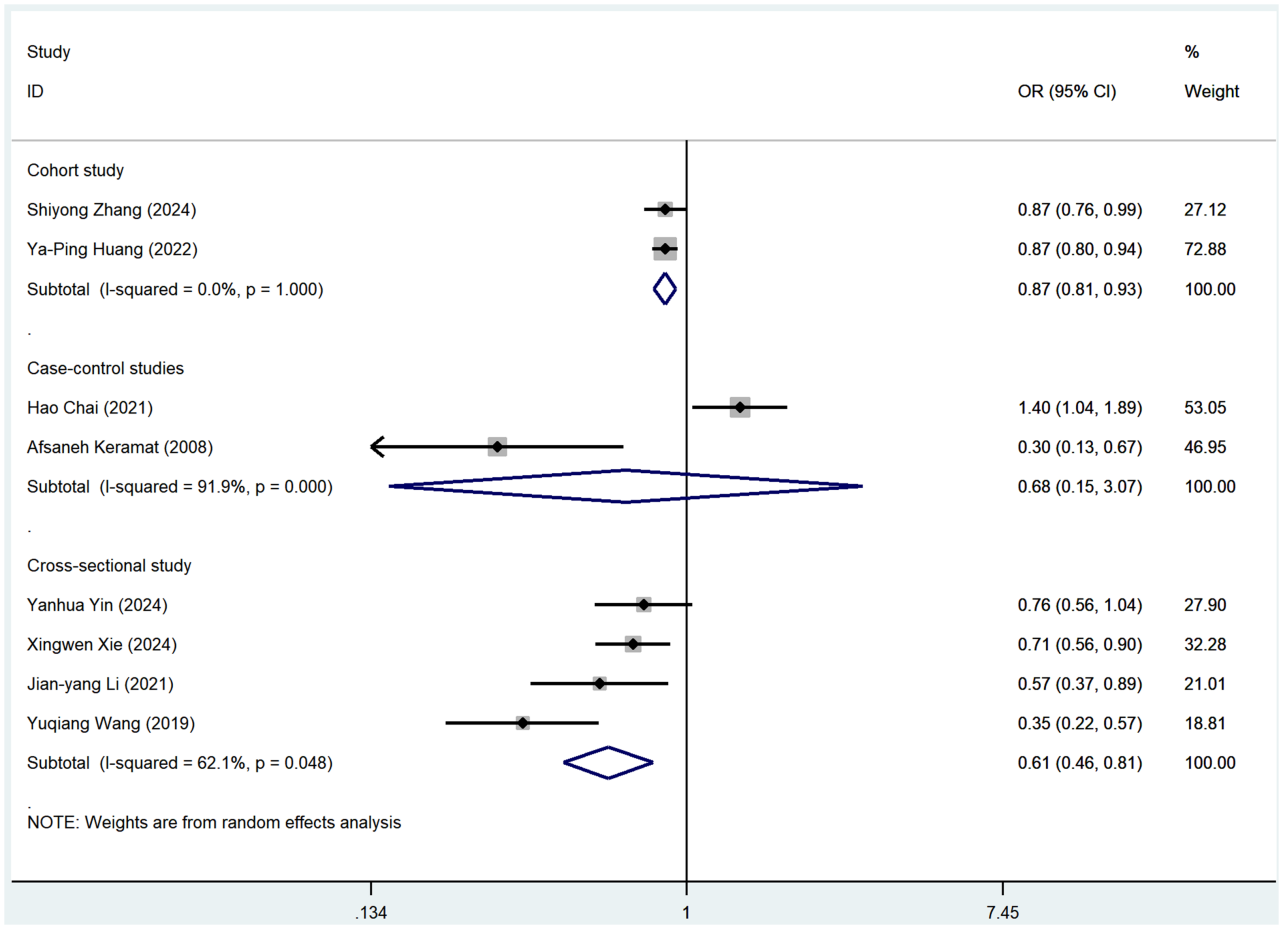
Subgroup analysis of different study types regarding tea consumption.

Subgroup analyses were conducted based on the frequency of coffee and tea consumption (Table 2; Supplementary file S5). For coffee, high-frequency consumption (defined as more than one cup per day) was associated with a slightly greater reduction in osteoporosis risk (OR = 0.83, 95% CI: 0.74–0.93, *p* = 0.001) than low-frequency consumption (defined as less than one cup per day), which showed no statistically significant reduction (OR = 0.86, 95% CI: 0.68–1.07, *p* = 0.171). Similarly, for tea consumption, high-frequency drinkers (defined as consuming tea more than four times per week) had a slightly stronger protective effect against osteoporosis (OR = 0.73, 95% CI: 0.62–0.86, *p* = 0.005) than low-frequency drinkers (OR = 0.82, 95% CI: 0.70–0.97, *p* = 0.111). To further mitigate potential bias risks associated with various study designs, we conducted subgroup analyses of coffee and tea consumption across three types of studies: cohort, case–control, and cross-sectional (Figures 4, 5). In cohort studies, coffee consumption exhibited low heterogeneity (OR = 0.78, 95% CI: 0.69–0.88, *I*^2^ = 0.0%, *p* = 0.320), and tea consumption also showed no significant heterogeneity among studies (OR = 0.87, 95% CI: 0.81–0.93, I^2^ = 0%, *p* = 1.000). In case–control studies, coffee consumption was not assessed for heterogeneity due to a single study (OR = 1.30, 95% CI: 0.74–2.30), but tea consumption had a high level of heterogeneity (OR = 0.68, 95% CI: 0.15–3.07, I^2^ = 91.9%, *p* = 0.000). Cross-sectional studies revealed low heterogeneity for coffee consumption (OR = 0.72, 95% CI: 0.62–0.85, I^2^ = 30.1%, *p* = 0.188) and a moderate level of heterogeneity for tea consumption (OR = 0.61, 95% CI: 0.46–0.81, *I*^2^ = 62.1%, *p* = 0.048).

**Table 2:** Subgroup analysis of osteoporosis caused by coffee or tea consumption.

Subgroups studies	Included study	OR 95%CI	Heterogeneity	
			*I*^2^ (%)	*p*-values
Frequency of coffee consumption				
Low coffee consumption (≤1 cup/day)	3	0.86 (0.68–1.07)	0	0.944
High coffee consumption (≥1 cup/day)	3	0.83 (0.74–0.93)	0	0.384
Frequency of tea consumption				
Low tea consumption (less than four times per week)	3	0.82 (0.70–0.97)	54.5	0.111
High tea consumption (more than four times per week)	5	0.73 (0.62–0.86)	72.9	0.005

### Publication bias

**Figure 6:**
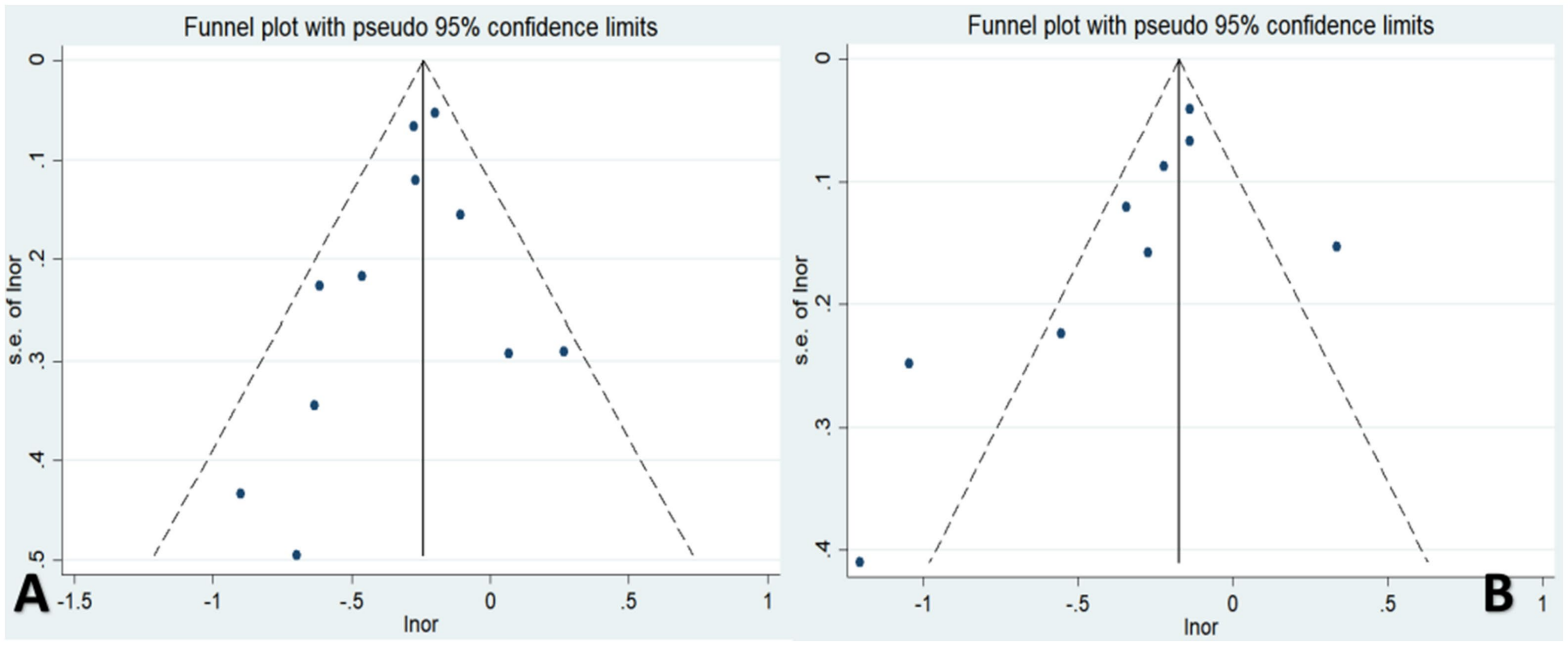
Publication bias of the risk of osteoporosis associated with coffee or tea consumption **(A,B)**.

Visual inspection of the funnel plots and Egger’s regression tests showed no significant evidence of publication bias for either coffee (*p* = 0.132) or tea (*p* = 0.328) consumption with respect to the risk of osteoporosis (Figure 6).

## Discussion

### Main findings

This meta-analysis, comprising 14 observational studies with 562,838 participants, evaluated the relationship between coffee and tea consumption and risk of osteoporosis. Our preliminary findings suggest that participants who regularly consume coffee and tea may have a lower risk of developing osteoporosis than non-consumers. Specifically, coffee drinkers showed a risk reduction of approximately 21%, and tea drinkers showed a risk reduction of approximately 25%. These findings tentatively imply that moderate daily consumption of coffee and tea can positively affect bone health and potentially reduce the risk of osteoporosis. It is noteworthy that the overall results of subgroup analyses by study design are consistent with the conclusions drawn. Although the subgroup analyses of the two case–control studies did not yield statistically significant results, this may be attributed to their small sample sizes or conflicting outcomes among individual studies. However, the impact on the overall results is minimal. However, it is important to note that this conclusion is based solely on the data available in the current study. The true relationship may be influenced by various factors such as individual genetic differences, lifestyle choices, dietary habits, and other potential confounders. Therefore, further investigation through larger-scale, multicenter, and more rigorously designed studies is necessary to confirm and validate the association between coffee and tea consumption and the reduction in osteoporosis risk.

### Interpretation of findings

Previous meta-analyses have explored the link between tea consumption and osteoporosis risk (15), with most studies showing a protective effect. However, because the outcomes considered here included BMD and osteoporotic fractures, we caution that the observed associations may not directly reflect a reduction in osteoporosis risk. However, the quality and heterogeneity of the studies included in these analyses varied, limiting the strength of the conclusions. For instance, one study found a negative correlation between tea consumption and osteoporosis risk, but did not observe differences in risk based on tea consumption frequency, likely because of the small sample size (36). Regarding coffee consumption, only one previous meta-analysis investigated its association with osteoporosis risk; however, its small sample size (only four studies) limited the robustness of its findings (16). This analysis did not show significant effects related to varying frequencies of coffee consumption, possibly due to the inclusion of osteoporotic fractures as an outcome rather than a direct osteoporosis diagnosis. In contrast, our study controlled for specific outcome variables and analyzed coffee and tea consumption at different frequencies, revealing a clear benefit of long-term consumption in reducing osteoporosis risk.

However, the mechanisms by which coffee affects osteoporosis are not yet fully understood. Although the effects of caffeine on bone health are controversial, some studies have suggested that low caffeine concentrations inhibit osteoclastogenesis and improve osteoporosis outcomes (37). However, higher concentrations have been shown to disrupt bone formation and increase the risk (14). Coffee also contains other bioactive compounds, such as flavonoids and potassium, which have positive effects on bone health through their anti-inflammatory and antioxidant properties (38, 39). Coffee is rich in phenolic compounds with osteoprotective potential, including chlorogenic acid (CGA). Polyphenols in CGA can stimulate osteoblast differentiation via the BMP-2/Wnt signaling pathway (40) and inhibit osteoclast genesis by regulating RANKL/OPG (41). Maillard reaction products (MRPs) formed during coffee roasting exhibit strong antioxidant, antibacterial, and anti-inflammatory properties. However, research suggests that advanced glycation end-products from the Maillard reaction may reduce bone mechanical properties and disrupt bone metabolism (42, 43), potentially affecting bone health. Further clinical studies are required to clarify the precise effects of MRPs on bone quality. Despite the potential synergistic effects of these compounds in reducing the risk of osteoporosis, their specific mechanisms require further investigation.

Tea, particularly its polyphenolic compounds, shows promise in protecting against osteoporosis. Studies have demonstrated that tea polyphenols improve bone microstructure, increase bone density, and modulate gut microbiota, thereby contributing to better bone health (44). Green tea catechins such as EGCG (Epigallocatechin gallate) have been shown to improve osteoblast and osteoclast activity, suggesting that tea consumption may prevent osteoporosis through multiple mechanisms (45). However, the potential adverse effects of tea on bone health, due to the complexity of its components, require further investigation.

### Implications and limitations

Our meta-analysis highlights the protective role of both coffee and tea in reducing osteoporosis risk, emphasizing the potential of a moderate, balanced diet in preventing osteoporosis. However, this study had several limitations. While controlling for confounders, the multivariable regression analyses used in most of the included studies did not provide deeper insights into sex differences or specific dosage effects. Future research should address these aspects to better understand the influence of coffee and tea consumption on the risk of osteoporosis. Furthermore, while the results of subgroup analyses align with the overall findings, the inherent heterogeneity in studies of various types necessitates caution in interpreting the results. In addition, this study has certain limitations in exploring the impact of coffee and related beverages on osteoporosis. It focused primarily on the general effects of coffee without considering the differences in components between various coffee varieties, such as Robusta and Arabica, and their specific effects on bone health. These coffee varieties differ significantly in the content of caffeine and bioactive compounds, which may lead to different mechanisms and varying effects on osteoporosis. Furthermore, this study did not address various coffee preparation methods, such as espresso, Turkish coffee, and filter coffee, which can alter the bioactive components in coffee and consequently influence bone health outcomes. Additionally, the research did not examine various tea beverages, including green, black, and oolong tea, which are rich in beneficial compounds and may have osteoporosis-related effects, either similar to or different from those of coffee. Future research exploring the relationship among coffee varieties, preparation methods, and tea beverages could provide a clearer understanding of their impact on osteoporosis and contribute to a more robust research framework.

## Conclusion

This meta-analysis demonstrated that daily coffee consumption exceeding one cup significantly reduced the risk of osteoporosis. However, no statistically significant effect was observed for low-frequency coffee consumption, suggesting that low coffee doses may not effectively prevent osteoporosis. Drinking tea more than four times a week appeared to have a stronger protective effect against osteoporosis, although the precise dosage was not clearly defined. Overall, for individuals within a moderate intake range—approximately one cup of coffee per day and tea more than four times a week—the protective effect against osteoporosis was more pronounced. These findings support the notion that moderate coffee and tea consumption as part of a balanced diet can prevent osteoporosis. Further research is required to explore the underlying mechanisms and clarify the effects of specific consumption patterns and dosages.

## Data Availability

The original contributions presented in the study are included in the article/Supplementary material, further inquiries can be directed to the corresponding author.
