# Supplementary figures and images for: Coffee and tea consumption on the risk of osteoporosis: a meta-analysis
Source: Front Nutr. 2025 Mar 4;12:1559835. doi: 10.3389/fnut.2025.1559835 (PMC11913691; doi:10.3389/fnut.2025.1559835)

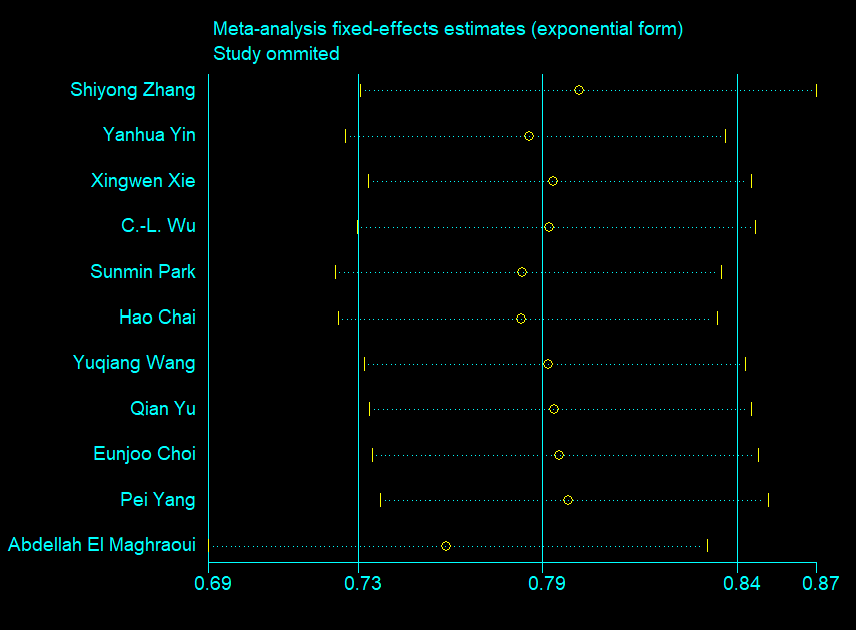

Supplement: Supplementary file 1 [file Image_1.tif]

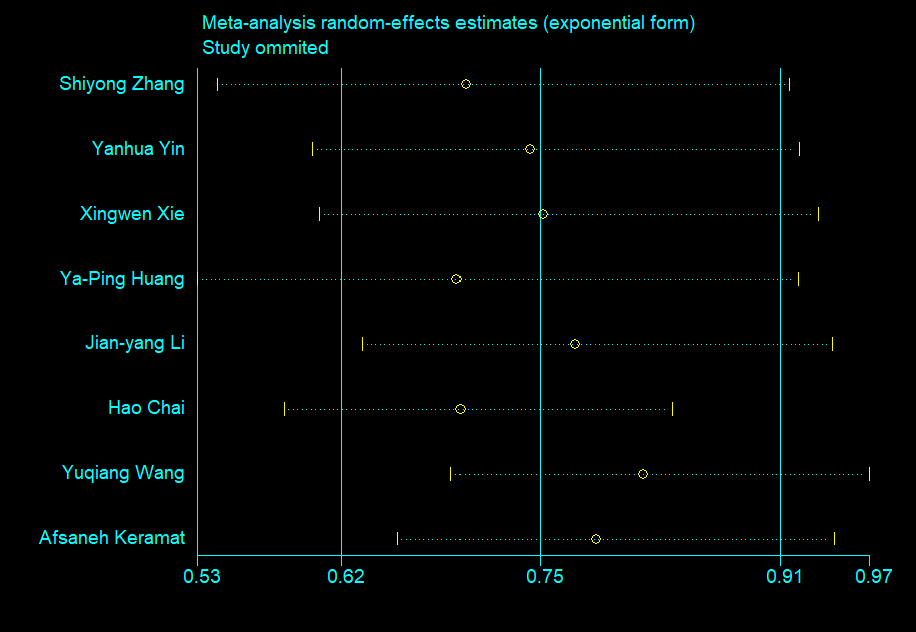

Supplement: Supplementary file 2 [file Image_2.tif]

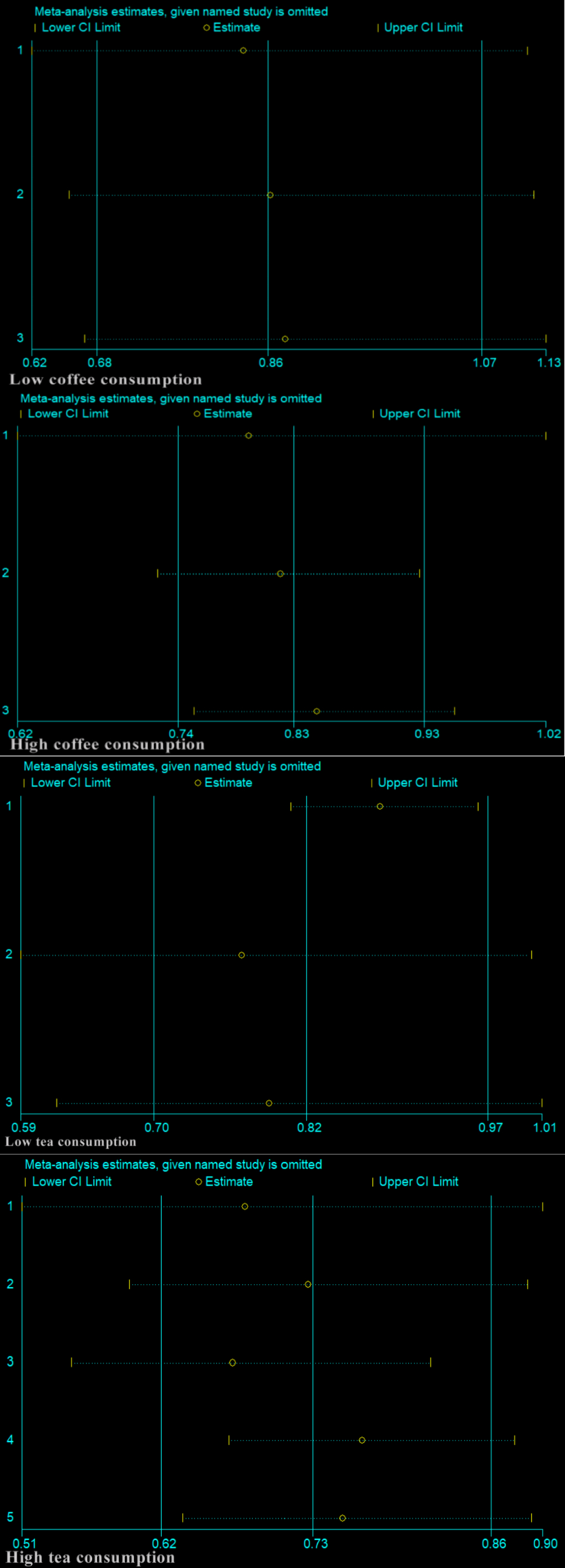

Supplement: Supplementary file 3 [file Image_3.tif]
