# Supplementary material for: Coffee and tea consumption on the risk of osteoporosis: a meta-analysis
Source: Front Nutr. 2025 Mar 4;12:1559835. doi: 10.3389/fnut.2025.1559835 (PMC11913691; doi:10.3389/fnut.2025.1559835)
Supplement: Supplementary file 5 [file Table_2.docx]

**The retrieval strategies and retrieval results of each database are shown in Tables 1-3**.

Table 1: PubMed 2024.11.5

| No. | Content | Result |
| --- | --- | --- |
| #1 | Search: "Osteoporosis"[Mesh] Sort by: Most Recent | 65803 |
| #2 | Search: ((Osteoporosis[Title/Abstract]) OR (Osteoporos*[Title/Abstract])) OR (Bone Loss*[Title/Abstract]) Sort by: Most Recent | 118900 |
| #3 | Search: ("Osteoporosis"[Mesh]) OR (((Osteoporosis[Title/Abstract]) OR (Osteoporos*[Title/Abstract])) OR (Bone Loss*[Title/Abstract])) Sort by: Most Recent | 134348 |
| #4 | Search: "Tea"[Mesh] Sort by: Most Recent | 14077 |
| #5 | Search: tea[Title/Abstract] Sort by: Most Recent | 42849 |
| #6 | Search: "Coffee"[Mesh] Sort by: Most Recent | 8860 |
| #7 | Search: ((coffee[Title/Abstract]) OR (coffee*[Title/Abstract])) OR (Espresso[Title/Abstract]) Sort by: Most Recent | 20763 |
| #8 | Search: ((("Tea"[Mesh]) OR (tea[Title/Abstract])) OR ("Coffee"[Mesh])) OR (((coffee[Title/Abstract]) OR (coffee*[Title/Abstract])) OR (Espresso[Title/Abstract])) Sort by: Most Recent | 61563 |
| #9 | Search: (("Osteoporosis"[Mesh]) OR (((Osteoporosis[Title/Abstract]) OR (Osteoporos*[Title/Abstract])) OR (Bone Loss*[Title/Abstract]))) AND (((("Tea"[Mesh]) OR (tea[Title/Abstract])) OR ("Coffee"[Mesh])) OR (((coffee[Title/Abstract]) OR (coffee*[Title/Abstract])) OR (Espresso[Title/Abstract]))) Sort by: Most Recent | 396 |

Table 2 Embase 2024.11.5

| No. | Content | Result |
| --- | --- | --- |
| #1 | 'osteoporosis'/mj | 64011 |
| #2 | 'osteoporosis'/exp OR osteoporosis:ab,ti OR osteoporos*:ab,ti OR 'bone loss*':ab,ti | 222133 |
| #3 | #1 OR #2 | 222133 |
| #4 | 'tea'/mj | 9929 |
| #5 | tea:ab,ti OR teas:ab,ti | 52952 |
| #6 | #4 OR #5 | 53308 |
| #7 | 'coffee'/mj | 7660 |
| #8 | coffee:ab,ti OR coffee*:ab,ti OR espresso:ab,ti | 25130 |
| #9 | #7 OR #8 | 25590 |
| #10 | #6 OR #9 | 74108 |
| #11 | #3 AND #10 | 607 |

Table 3 Cochran Library 2024.11.5

| No. | Content | Result |
| --- | --- | --- |
| #1 | MeSH descriptor: [Osteoporosis] this term only | 3231 |
| #2 | (Osteoporosis):ti,ab,kw OR (Osteoporos*):ti,ab,kw OR (Bone Loss*):ti,ab,kw | 22469 |
| #3 | #1 or #2 | 22469 |
| #4 | MeSH descriptor: [Tea] this term only | 719 |
| #5 | (tea):ti,ab,kw OR (teas):ti,ab,kw | 5144 |
| #6 | MeSH descriptor: [Coffee] this term only | 555 |
| #7 | (coffee):ti,ab,kw OR (coffee*):ti,ab,kw OR (Espresso):ti,ab,kw | 2375 |
| #8 | #4OR#5OR#6OR#7 | 6882 |
| #9 | #3AND#8 | 44 |
